# Hessian fly larval feeding triggers enhanced polyamine levels in susceptible but not resistant wheat

**DOI:** 10.1186/s12870-014-0396-y

**Published:** 2015-01-16

**Authors:** Subhashree Subramanyam, Nagesh Sardesai, Subhash C Minocha, Cheng Zheng, Richard H Shukle, Christie E Williams

**Affiliations:** Department of Agronomy, Purdue University, West Lafayette, IN 47907 USA; Department of Biological Sciences, University of New Hampshire, Durham, NH 03824 USA; Department of Statistics, Purdue University, West Lafayette, IN 47907 USA; Department of Entomology, Purdue University, West Lafayette, IN 47907 USA; USDA-ARS Crop Production and Pest Control Research Unit, West Lafayette, IN 47907 USA; Present address: Dow AgroSciences LLC, Indianapolis, IN 46268 USA; Present address: Novartis Pharmaceuticals Corporation, East Hanover, NJ 07936 USA

**Keywords:** Polyamines, Wheat, Hessian fly, Compatible, Incompatible, RT-qPCR, Odc, Samdc, Spds

## Abstract

**Background:**

Hessian fly (*Mayetiola destructor*), a member of the gall midge family, is one of the most destructive pests of wheat (*Triticum aestivum*) worldwide. Probing of wheat plants by the larvae results in either an incompatible (avirulent larvae, resistant plant) or a compatible (virulent larvae, susceptible plant) interaction. Virulent larvae induce the formation of a nutritive tissue, resembling the inside surface of a gall, in susceptible wheat. These nutritive cells are a rich source of proteins and sugars that sustain the developing virulent Hessian fly larvae. In addition, on susceptible wheat, larvae trigger a significant increase in levels of amino acids including proline and glutamic acid, which are precursors for the biosynthesis of ornithine and arginine that in turn enter the pathway for polyamine biosynthesis.

**Results:**

Following Hessian fly larval attack, transcript abundance in susceptible wheat increased for several genes involved in polyamine biosynthesis, leading to higher levels of the free polyamines, putrescine, spermidine and spermine. A concurrent increase in polyamine levels occurred in the virulent larvae despite a decrease in abundance of *Mdes-odc* (ornithine decarboxylase) transcript encoding a key enzyme in insect putrescine biosynthesis. In contrast, resistant wheat and avirulent Hessian fly larvae did not exhibit significant changes in transcript abundance of genes involved in polyamine biosynthesis or in free polyamine levels.

**Conclusions:**

The major findings from this study are: (i) although polyamines contribute to defense in some plant-pathogen interactions, their production is induced in susceptible wheat during interactions with Hessian fly larvae without contributing to defense, and (ii) due to low abundance of transcripts encoding the rate-limiting ornithine decarboxylase enzyme in the larval polyamine pathway the source of polyamines found in virulent larvae is plausibly wheat-derived. The activation of the host polyamine biosynthesis pathway during compatible wheat-Hessian fly interactions is consistent with a model wherein the virulent larvae usurp the polyamine biosynthesis machinery of the susceptible plant to acquire nutrients required for their own growth and development.

**Electronic supplementary material:**

The online version of this article (doi:10.1186/s12870-014-0396-y) contains supplementary material, which is available to authorized users.

## Background

Polyamines are ubiquitous, low-molecular-weight aliphatic polycations that play a vital role in regulating gene expression, signal transduction, ion-channel function, DNA and protein synthesis as well as cell proliferation and differentiation [1]. They scavenge reactive oxygen species thereby protecting DNA, proteins, and lipids from oxidative damage [2]. In plants, the most common polyamines are diamine putrescine, triamine spermidine, and tetramine spermine [3]. They occur either in free form or as conjugates bound to phenolic acids and low molecular weight compounds. Due to their positive charge, polyamines interact with negatively charged macromolecules such as proteins and nucleic acids leading to the stabilization of these molecules under stress conditions [4,5].

**Figure 1:**
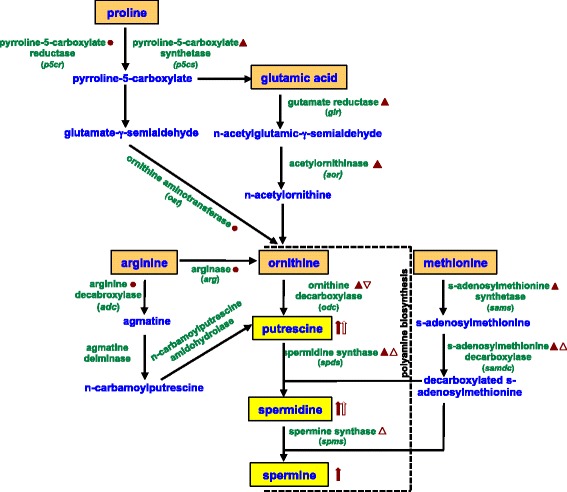
**Ornithine and polyamine biosynthesis pathway.** The principle pathway of ornithine and polyamine biosynthesis is shown along with a summary of key findings from the current study. Change in abundance of transcripts in susceptible wheat plants is indicated by solid triangles and in virulent Hessian fly larvae as open triangles, compared to controls. Triangles pointing up or down indicate increase or decrease, respectively, in transcript abundance quantified by RT-qPCR. Solid circles indicate transcripts that are either transiently expressed in only one time-point or do not differ significantly from control levels in wheat tissue. Solid block-style arrows indicate polyamine levels in susceptible wheat plants and open block-style arrows indicate polyamine levels in virulent Hessian fly larvae. Arrows pointing up indicate increased levels of polyamines in infested tissue compared to uninfested controls or in virulent larvae compared to avirulent larvae.

In plants, the first step in polyamine biosynthesis is the formation of putrescine from either ornithine or arginine (Figure 1). Ornithine is converted directly into putrescine by ornithine decarboxylase (ODC). Arginine can be converted into ornithine by arginase, or can take a longer route whereby it is converted to agmatine by arginine decarboxylase (ADC), then to *n*-carbamoylputrescine by agmatine deiminase and finally into putrescine by *n*-carbamoylputrescine amidohydrolase. Putrescine subsequently receives an aminopropyl moiety from decarboxylated S-adenosylmethionine (SAMDC) via spermidine synthase (SPDS) to produce spermidine; and spermine is then generated by a second aminopropyl transfer by spermine synthase (SPMS) [6].

Involvement of polyamines in plant disease resistance has been extensively reviewed [7-9]. Polyamine catabolism produces H_2_O_2_, which plays a role in plant defense by contributing to the hypersensitive response [9-11] that acts against different biotic stressors like fungi, bacteria and viruses [12,13]. Some examples of polyamines associated with plant defense include castor (*Ricinus communis*) against *Fusarium oxysporum f. sp. ricini* [14], *Arabidopsis* against *Pseudomonas syringae* [15] and tobacco in response to inoculation with Tobacco Mosaic Virus (TMV) [16]. Monocots also respond with increased polyamine levels during defense against microbial pathogens. In an incompatible interaction between barley and powdery mildew (*Blumeria graminis f. sp. hordei*), levels of free and conjugated spermidine and putrescine as well as activity of ODC, ADC and SAMDC enzymes increased, three days after inoculation [17].

Despite documented changes of plant polyamine levels in response to various microbial pathogens, limited information is available on their involvement in plant-pest interactions. Increased abundance of polyamines during plant resistance has been reported for interactions between sweet pepper and leafminer [18] and during tolerance in *Nicotiana attenuata* attacked by mirid bug [19] and triticale infested by aphids [20]. One proposed function in plant defense is that phenolic polyamines block glutamatergic neuromuscular junctions resulting in paralysis of insect skeletal muscles [21]. Other defense mechanisms associated with increased polyamine abundance include spider mite-induced plant volatiles that attract carnivorous natural enemies to lima bean [22] and disrupted settling of bird cherry-oat aphids on triticale [20].

Hessian fly (*Mayetiola destructor*), a member of the gall midge family (Cecidomyiidae) is a destructive insect pest of wheat (*Triticum aestivum*) causing significant economic losses worldwide [23]. This insect is an obligate parasite that must receive all of its nutrition from the host plant. Following egg hatch, the first-instar Hessian fly larvae crawl down the leaf blade to the base (crown) of the wheat plant and attempt to establish sustained feeding sites. Probing by the larvae results in either an incompatible (avirulent larvae, resistant plant) or a compatible (virulent larvae, susceptible plant) interaction.

Resistance of wheat to Hessian fly attack is achieved through the action of any of 35 distinct resistance genes (*H1-H34* plus *Hdic*) identified so far [24-27]. Gene-for-gene interaction [28] is thought to occur when a larval salivary gene product is recognized by a wheat resistance gene product [29]. The resulting incompatible interactions are characterized by expression of defense response genes [30,31], accumulation of feeding deterrent proteins [32,33], and changes in surface wax composition [34] as well as host-cell permeability that aids in delivery of these substances [35] and ultimately leads to larval death.

During compatible interactions, salivary effectors from virulent larvae suppress wheat defense responses leading to susceptibility, which allows the insect to complete its life cycle [36,37]. Within three to four days of larval attack, the virulent larvae alter host metabolic pathways [38] resulting in differentiation of a nutritive tissue at the feeding site, which is believed to provide the larvae a diet rich in essential nutrients [39]. These physiological changes are accompanied by a shift from carbon-containing compounds to elevated levels of nitrogen-containing compounds with corresponding changes in transcript levels of genes involved in glycolysis, the pentose phosphate pathway, and the tricarboxylic acid cycle [38]. The carbon/nitrogen shift may provide better nutrition for insect development. In addition, a significant increase in levels of certain amino acids, including, proline, glycine, serine, tyrosine and glutamic acid, were observed in nutritive tissue [40]. Proline, glycine, serine and tyrosine are ‘conditionally essential’ amino acids, meaning they become essential only when the organism faces periods of extreme stress where the physiological need exceeds the organism’s ability to produce. Although methionine abundance does not increase in compatible interactions, it is an essential amino acid that cannot be synthesized de novo by an animal and must be supplied in its diet. The demand for amino acids expands beyond the essential set to the conditionally essential set in rapidly developing insect tissues [41]. Therefore, these nutrients must be supplied exogenously through diet. Proline, glutamic acid and methionine enter the ornithine biosynthesis pathway, eventually leading to the production of polyamines.

The present study focuses on the polyamine biosynthesis pathways in both wheat and Hessian fly larvae during compatible (susceptible plant) and incompatible (resistant plant) interactions. We addressed two hypotheses. The first hypothesis was that wheat production of polyamines would increase as a component of its defense response against attack by Hessian fly larvae. This assumption was based on numerous reports of polyamine accumulation in response of resistant plants to biotic stresses [42]. The second hypothesis was that the polyamine biosynthetic pathway would be highly up-regulated in virulent Hessian fly larvae to support the rapid growth processes driven by gene transcription and translation, as is the case in organisms ranging from mammals to bacteria [42]. We report differences in polyamine levels as well as in the transcript abundance of key genes involved in biosynthesis of polyamines in susceptible and resistant wheat plants during response to feeding by Hessian fly larvae. In addition, polyamine levels and biosynthetic pathway were monitored in virulent Hessian fly larvae. The implications of increased polyamines as an additional source of nutrition leading to development of the virulent Hessian fly larvae are discussed.

## Results

### Polyamine levels increase in susceptible wheat and virulent Hessian fly larvae

**Figure 2:**
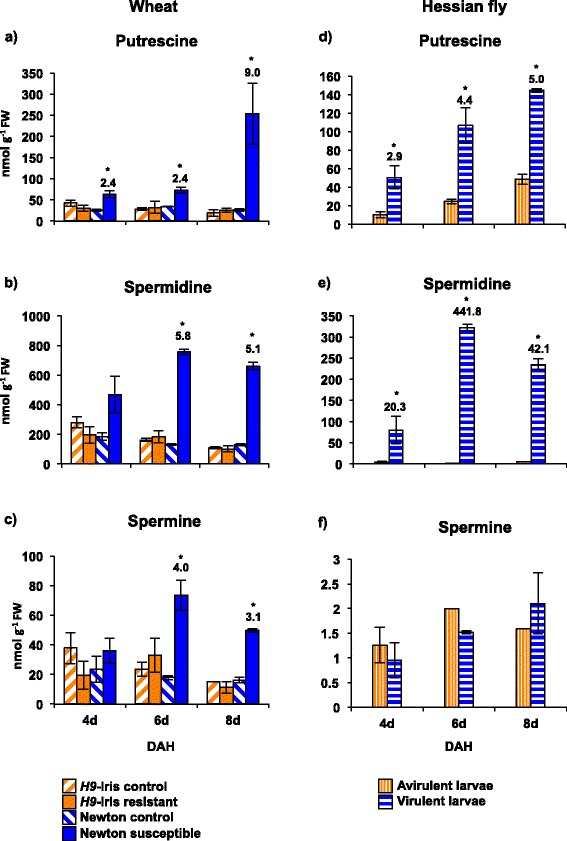
**Wheat and Hessian fly polyamine levels. Panels a**, **b**, and **c** show polyamine levels in *H9*-Iris (resistant, incompatible interaction) and Newton (susceptible, compatible interaction) wheat crown tissue (leaf 2) at the larval feeding site. **Panels d**, **e**, and **f** show levels in avirulent and virulent biotype L Hessian fly larvae feeding on *H9-*Iris and Newton wheat plants, respectively. Error bars represent mean ± SE of two independent biological replicates. Statistically significant (*p* < 0.05) differences in polyamine levels between infested and uninfested (control) wheat plants **(panels a, b, c)** and between virulent and avirulent larvae **(panels d, e, f)** are indicated by ‘*’ with fold-change values.

Metabolite profiling using HPLC detected differences in the free polyamine levels between resistant and susceptible wheat plants following Hessian fly (biotype L) larval attack (Figure 2a-c). In susceptible Newton wheat, putrescine concentration increased to more than two-fold (*p* = 0.005) at 4 and 6 DAH, and nine-fold (*p* = 0.01) by 8 DAH above levels in the uninfested control (Figure 2a). Spermidine and spermine levels did not increase significantly above control levels at 4 DAH in susceptible wheat (Figure 2b-c). However, they increased significantly in the susceptible wheat by 6 DAH (5.8-fold spermidine; 4-fold spermine, *p* < 0.0001), and then slightly decreased by 8 DAH (5.1-fold spermidine, *p* < 0.001; 3.1-fold spermine, *p* < 0.0001). In contrast, resistant *H9*-Iris wheat showed no change in any of the polyamine levels relative to the uninfested controls (Figure 2a-c).

Polyamine levels in virulent and avirulent Hessian fly larvae positively correlated with the levels observed in susceptible and resistant host plants over the time-course (Figure 2d-f). In virulent larvae feeding on the susceptible plants, putrescine levels increased from 3- to 5-fold between 4 and 8 DAH (*p* = 0.04) above levels in the avirulent larvae. Spermidine levels increased significantly in virulent larvae from 20-fold by 4 DAH (*p* = 0.01) to over 440-fold (*p* = 0.005) by 6 DAH (Figure 2e). This level decreased to 40-fold (Figure 2e) by 8 DAH (*p* < 0.0001). Spermine levels were low and did not vary significantly between the virulent and avirulent larvae (Figure 2f). In the avirulent larvae the levels of putrescine, spermidine and spermine remained unchanged showing no significant difference (*p* = 1) at all time-points (Figure 2d-f). In both, susceptible wheat and the virulent larvae, spermidine was by far the most abundant of the three polyamines investigated in the current study.

### Wheat polyamine pathway transcript abundance parallels polyamine levels

**Figure 3:**
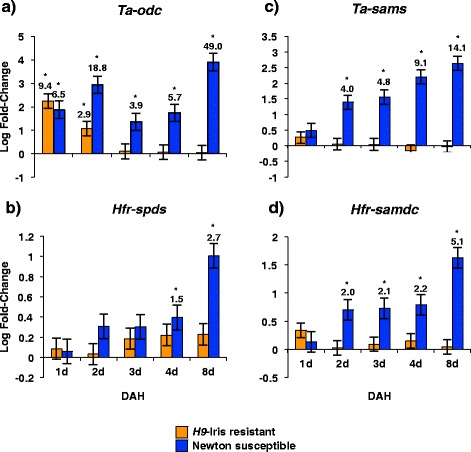
**Abundance of polyamine biosynthesis pathway transcripts in** ***H9-Iris*** **and Newton wheat infested with biotype L Hessian fly larvae.** Transcript levels of **a)** *Ta-odc*, **b)** *Hfr-spds*, **c)** *Ta-sams*, and **d)** *Hfr-samdc* in crown tissue (leaf 2) quantified by RT-qPCR. Values are the log fold-change ± SE of infested compared to uninfested control (baseline of 0) plants. Statistically significant (*p* < 0.05) differences are indicated by ‘*’ with linear fold-change values.

The biosynthesis of putrescine, spermidine and spermine from amino acids involves several enzymatic steps. To determine which of the genes in the polyamine biosynthesis pathway are activated by Hessian fly infestation we carried out RT-qPCR expression studies (Figure 3). In susceptible Newton wheat infested with biotype L, transcripts encoding ornithine decarboxylase (*Ta-odc)*, s-adenosylmethionine synthetase (*Ta-sams*) and s-adenosylmethionine decarboxylase *(Hfr-samdc*) were significantly responsive over time from 2 through 8 DAH compared to the uninfested controls. While arginine decarboxylase (*Ta-adc*) did not show an increase in transcript abundance (data not shown), spermidine synthase (*Hfr-spds*) showed a small but significant increase only at later times (Figure 3b). In contrast, in the resistant *H9*-Iris wheat line only transcripts for *Ta-odc* accumulated to significantly higher levels than the uninfested control following attack by the avirulent larvae (Figure 3a). Transcript levels of polyamine oxidase (*Ta-pao*), involved in the catabolism of polyamines did not show any change in either susceptible or resistant wheat (data not shown). Transcriptional profiling studies carried out in other wheat genotypes infested with either a different Hessian fly biotype or harboring a different *R* gene (*vH9* on *H9-*Iris wheat, Additional file 1; *vH13* on *H13*-wheat, Additional file 2) yielded very similar patterns of expression with significant accumulation of polyamine pathway transcripts during compatible interactions.

### Transcripts encoding enzymes for amino acid utilization in ornithine biosynthesis accumulate in susceptible wheat

**Figure 4:**
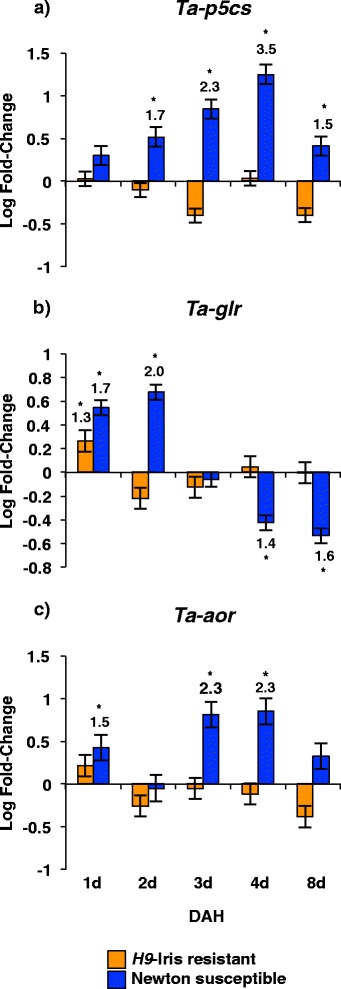
**Abundance of ornithine biosynthesis pathway transcripts in** ***H9-Iris*** **and Newton wheat infested with biotype L Hessian fly larvae.** Transcript levels of **a)** *Ta-p5cs*, **b)** *Ta-glr*, and **c)** *Ta-aor* from crown tissue (leaf 2) quantified by RT-qPCR. Values are the log fold-change ± SE of infested compared to the uninfested control plants. Statistically significant (*p* < 0.05) differences are indicated by ‘*’ with linear fold-change values.

Expression (RT-qPCR) studies revealed increased abundance of transcripts encoding enzymes catalyzing the conversion of the precursor amino acids proline and glutamic acid to ornithine (Figure 4). Transcripts for genes encoding pyrroline-5-carboxylate synthetase (*Ta-p5cs*), glutamate reductase (*Ta-glr*) and acetylornithinase (*Ta-aor*) were most responsive in the susceptible Newton wheat (Figure 4), whereas transcripts of pyrroline-5-carboxylate reductase (*Ta-p5cr*), arginase (*Ta-arg*), and ornithine aminotransferase (*Ta-oat*), showed a minimal transient response (Additional file 3). A similar expression profile was observed in other wheat genotypes infested with different fly biotypes also resulting in compatible interactions (Additional files 4 and 5). However, unlike the *H9*-wheat, the *H13*-wheat transcript abundance increased for *Ta-oat* and decreased for *Ta-glr.*

### Activity of s-adenosylmethionine decarboxylase (SAMDC) increases in susceptible wheat after Hessian fly larval attack

**Figure 5:**
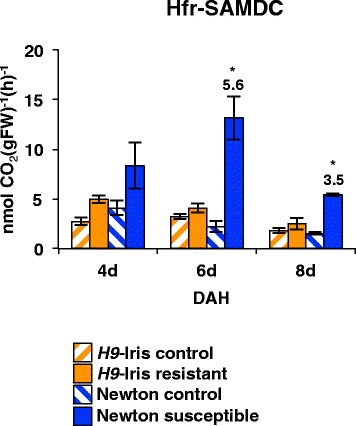
**Specific activity of wheat Hfr-SAMDC (s-adenosyl methionine decarboxylase) in** ***H9-Iris*** **and Newton wheat infested with biotype L Hessian fly larvae.** Hfr-SAMDC enzymatic activity was measured in wheat crown tissue (leaf 2). Data are presented as mean ± SE. Statistically significant (*p* < 0.05) differences between infested and uninfested control are indicated by ‘*’ with fold-change values.

Increase in *Hfr-samdc* transcript abundance (Figure 3d) resulted in higher Hfr-SAMDC enzyme activity in the susceptible wheat line after Hessian fly attack. Significantly higher levels of Hfr-SAMDC activity were detected in the infested susceptible plants than in uninfested controls at 6 (5.6-fold, *p* = 0.0032) and 8 (3.5-fold, *p* = 0.0256) DAH (Figure 5). Although *Hfr-SAMDC* transcripts were significantly higher at 4 DAH in susceptible wheat (2.2-fold, *p* < 0.001, Figure 3), significant increases in Hfr-SAMDC enzyme activity were not detected until later. At no time did Hfr-SAMDC activity significantly differ (*p* > 0.4) between the resistant and their uninfested control plants (Figure 5).

### Annotation and phylogenetic reconstruction of *M. destructor* genes involved in synthesis of polyamines

To identify Hessian fly polyamine biosynthesis genes for use in carrying out transcript analysis, these genes were annotated from the Hessian fly genome assembly. We successfully annotated near full-length cDNA sequence for *Mdes*-*odc*, *Mdes-spds* and *Mdes-spms* genes and a partial cDNA sequence for *Mdes*-*samdc* (Additional file 6: Table S1). The sequences for all four genes were highly similar to their respective orthologs annotated from the *Aedes aegypti* genome. We were unable to annotate s-adenosylmethionine synthetase from the Hessian fly genome assembly. The annotated genes were cloned and sequenced to validate the Gbrowse annotated sequences. Phylogenetic reconstructions grouped the genes with their respective orthologs from other insect species verifying that the correct Hessian fly genes were identified for use in expression studies (Additional file 7).

### Virulent and avirulent Hessian fly larvae exhibit differential expression of polyamine biosynthesis pathway genes

**Figure 6:**
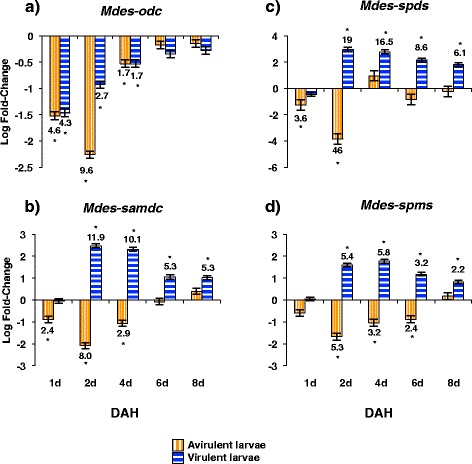
**Abundance of Hessian fly larval transcripts for polyamine biosynthesis.** Transcript levels of **a)** *Mdes-odc*, **b)** *Mdes-samdc*, **c)** *Mdes-spds*, and **d)** *Mdes-spms* were quantified by RT-qPCR. Values are the log fold-change ± SE for avirulent and virulent Hessian fly larvae that have fed on host plants compared to neonate larvae (collected on the day of egg hatch; baseline of 0) that had not fed on plants. Statistically significant (*p* < 0.05) differences are indicated by ‘*’ with linear fold-change values.

As polyamine levels of susceptible wheat increased, so did polyamine levels in the virulent Hessian fly larvae. To ascertain whether increased larval polyamine levels were caused by activation of polyamine pathway genes in the larvae or whether larval polyamines were plant-derived we carried out RT-qPCR studies to look at expression of *Mdes-odc*, *Mdes-samdc*, *Mdes-spds* and *Mdes-spms* genes in the virulent and avirulent Hessian fly larvae. Expression levels were compared to those in neonate larvae that had never fed on a wheat plant. ODC is considered the rate-determining enzyme in polyamine biosynthesis; however, transcripts for *Mdes-odc* were significantly less abundant in virulent larvae than in the neonate larvae (Figure 6a). In contrast, transcripts for the other three genes increased greatly (Figure 6b-d) in abundance 2–4 DAH (once the virulent larvae had established feeding sites), indicating an increased capacity, especially for spermidine production, through non-*Mdes-odc* entry points. The abundance of *Mdes-samdc*, *Mdes-spds* and *Mdes-spms* transcripts gradually decreased by 8 DAH in virulent larvae (Figure 6b-d). In the avirulent Hessian fly larvae, transcripts for all four genes under study were significantly lower at all stages of development as compared to the neonate larvae (Figure 6a-d).

### Inhibiting wheat ornithine decarboxylase enzyme activity limits Hessian fly larval growth

**Figure 7:**
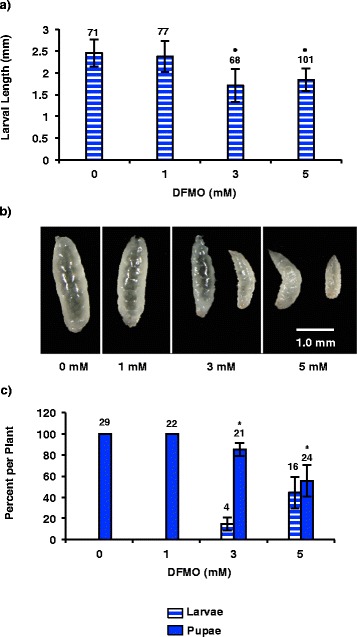
**Hessian fly larval responses to inhibition of wheat ODC activity with Difluoromethylornithine (DFMO). a)** Length of biotype L larvae (measured 7 DAH) feeding on susceptible Newton wheat plants that were pretreated with 1, 3 and 5 mM concentrations of DFMO to block wheat ODC activity. Data are represented as mean larval length ± SE for the respective number of larvae (given above each bar) measured for each treatment. Treatments showing statistically significant (*p* < 0.05) differences between DFMO-treated and untreated plants (0 mM DFMO) are indicated with ‘*’. **b)** Representative photomicrographs of biotype L Hessian fly larvae from each of the treatments. **c)** Mean percentage ± SE for the respective number (given above each bar) of insects for each treatment in larval (solid filled bars) or pupal stages (striped bars) on treated susceptible Newton wheat plants 18 DAH. Treatments showing statistically significant (*p* < 0.05) differences from the control (0 mM DFMO) are indicated with ‘*’.

To study the effects of limiting wheat polyamine production on virulent Hessian fly larval growth, we used DFMO to inhibit ODC enzymatic activity of the susceptible host plants. The larvae were prevented from coming into direct contact with the applied DFMO because the blade of the first leaf was painted with the inhibitor and allowed to dry before adult flies were released onto the plant. The eggs were oviposited on the second leaf blade ensuring lack of direct contact with the DFMO. Since peak abundance of most polyamines as well as the transcripts encoding the enzymes were observed between 4 and 8 DAH, larval length measurements were taken 7 DAH. The larvae growing on plants treated with 3 or 5 mM DFMO were significantly smaller (*p* < 0.0001) compared to larvae on untreated plants (Figure 7a-b). No significant difference (*p* = 0.4667) was seen in the size of larvae growing on plants treated with 1 mM DFMO. In addition, at concentrations of 3 and 5 mM DFMO the percentage of insects that had reached pupation 18 DAH was significantly lower (Figure 7c) indicating delayed larval development. Larvae inhabiting plants treated with 1 mM DFMO did not exhibit significant differences in pupation rate as compared to the control (Figure 7c).

## Discussion

The current study was undertaken to examine temporal changes in free polyamine abundance and expression of genes contributing to polyamine biosynthesis during wheat interactions with Hessian fly larvae. Key findings summarized in Figure 1 were: (i) susceptible wheat: increased levels of ornithine and polyamine biosynthesis gene transcripts plus higher SAMDC enzyme activity resulted in greater putrescine, spermidine and spermine abundance, (ii) resistant wheat: the polyamine pathway was unresponsive to Hessian fly attack, and (iii) virulent larvae: although putrescine and spermidine levels increased, transcripts encoding *Mdes-odc* (ODC is rate-limiting enzyme for polyamine biosynthesis in insects [43]) decreased in abundance. Irrespective of the wheat genotype or Hessian fly biotype used, these results were consistently observed in all compatible wheat-Hessian fly interactions.

Resistant plants exhibited no change in either the transcript levels of genes that encode enzymes for polyamine biosynthesis or in the levels of free polyamines. In contrast, the induction of wheat susceptibility resulted in increased polyamine production. Thus, our first hypothesis that polyamine production would increase as a component of wheat defense response against attack by Hessian fly larvae was not supported. Although higher polyamine levels are predominantly associated with induced plant resistance, their increase has occasionally been associated with susceptibility in cereals. Elevated spermidine levels (6- to 7-fold higher than controls) were observed in susceptible barley leaves that had “green islands” surrounding the infection sites of brown rust and powdery mildew fungi [44]. Likewise, stripe rust caused an increase in the polyamine content in a susceptible wheat cultivar [45]. Feeding by bird cherry-oat aphid (*Rhopalosiphum padi)* resulted in higher level of putrescine and spermidine in shoots of susceptible triticale cultivars [20].

Our earlier observations of a significant increase in susceptible wheat production of glutamic acid (l.61-fold), proline (4.79-fold), and alanine (2.18-fold) by day four following Hessian fly larval attack [40] suggest a linkage to the increase in polyamine production. In that study, small but significant increases in mRNA abundance for alanine aminotransferase and glutamine-dependent asparagine synthetase, lead to glutamic acid becoming the most abundant free amino acid produced at the larval feeding sites in susceptible wheat [40]. Building on that information, the current study showed increased abundance of transcripts for *Ta-p5cs*, *Ta-glr*, and *Ta-aor* in susceptible wheat indicating that at least part of the increased production of proline and glutamic acid is shunted into the polyamine pathway via ornithine. Further, the increased levels of *Ta-odc*, *Ta-sams*, *Hfr-samdc* and *Hfr-spds* transcripts, as well as increased abundance of all three free polyamines observed in susceptible crown tissue, provide evidence that the increased wheat polyamine synthesis is an integral part of the compatible interaction with Hessian fly larvae.

Generally, both ODC- and ADC-mediated polyamine biosynthesis is induced in plants as a response to biotic [7] and abiotic stresses [46]. However, induction of the ODC-mediated pathway seems to be the predominant mode of polyamine biosynthesis during plant biotic stress as compared to abiotic stress [47]. Our results showed a greater increase in *Ta-odc* transcripts (up to 49-fold) than *Ta-adc* transcripts (up to 2.2-fold), in susceptible wheat following Hessian fly attack, implicating ODC-mediated polyamine biosynthesis as the predominant entry into this pathway.

Resource manipulation of the host plant is a common life strategy for insects that are obligate parasites. The group of gall-forming insects, which includes the Hessian fly, uses an effector-based mechanism to reorient the physiology of the host, creating a sustainable environment that provides physical protection and quality nutrients [48,49]. Like amino acids, the pool of polyamines in an organism is maintained by *de novo* synthesis, exogenous supply through the diet or both [50]. Among other functions, polyamines are growth factors and are required to maintain metabolic processes in all organisms [51]. Several studies document benefits of dietary polyamines during insect development. One example showed increased larval survival and the rate of development for saw-toothed grain beetle (*Oryzaephilus surinamensis*) when putrescine was added to an artificial diet [52]. However, induction of host-plant polyamine production may benefit Hessian fly larvae in other ways. Like fungal pathogens that manipulate polyamine levels to maintain “green islands”, tissue in a juvenile and metabolically active state in an otherwise senescing cereal leaf [8], Hessian fly larvae require their host wheat plant to continue producing nutrients throughout their feeding stages. Hessian fly-infested susceptible wheat plants are known to be darker green than resistant or control plants [53] and thus the entire plant may represent a “green island”. Because polyamines offer some degree of protection against pathogen attack as well as oxidative, acidic and osmotic stresses [54], their increased production could benefit both the susceptible wheat plant and the virulent larvae. Susceptible wheat mounts a basal defense against Hessian fly larvae that includes production of molecules such as reactive oxygen species [55,56] and lectins [57,58] that have the capacity to damage the larval midgut when ingested. In resistant tobacco plants, in response to TMV infection, polyamine degradation by polyamine oxidase is a source of H_2_O_2_ leading to a hypersensitive response [59]. However, no increase in transcripts of wheat polyamine oxidase was observed in either compatible or incompatible interactions (data not shown) suggesting that polyamines are the terminal catabolic products that are utilized by the Hessian fly larvae. The contribution of polyamines to gut repair following injury [60], may help protect the midgut of virulent larvae from basal defenses since no visible damage was detected in the midgut of virulent larvae feeding on susceptible wheat [61].

Our expression profiling studies revealed low abundance of *Mdes-odc* transcripts in both virulent and avirulent larvae, which should limit the production of downstream polyamines. However, abundance of *Mdes-samdc*, *Mdes-spds*, and *Mdes-spms* transcripts increased significantly and so did polyamine abundance 2 DAH in the virulent Hessian fly larvae. Thus our second hypothesis, that the polyamine biosynthetic pathway would be highly up-regulated in virulent Hessian fly larvae to support the rapid growth processes driven by gene transcription and translation, was only partially supported. Since ODC is a rate-limiting enzyme in the conversion of ornithine to putrescine [50], the increasing levels of larval putrescine, which parallel the increasing levels in the host wheat plant, may be of plant origin.

The experiment utilizing DFMO to block wheat ODC activity (responsible for conversion of ornithine to putrescine) and thus decrease polyamine production, resulted in a significant decrease in larval size and rate of development, providing further evidence for a plant-derived source of polyamines in the virulent larvae. DFMO application to the first leaf before infesting with Hessian flies on the second leaf minimized the chances that the DFMO came in direct contact with either eggs or larvae. Although DFMO is systemically translocated in plants [62,63] and thus small amounts could be ingested by larvae, the effect of inhibiting larval ODC should be small since *Mdes-odc* transcript levels are already very low in larvae (Figure 6a). The objective of the experiment was to inhibit the plant ODC enzyme with DFMO to decrease the availability of putrescine for ingestion by the virulent Hessian fly larvae. Since these larvae were significantly smaller and exhibited delayed pupation compared to larvae on the control plants without DFMO, it appears that larval development was negatively affected by decreasing levels of putrescine in the host plant.

## Conclusions

**Figure 8:**
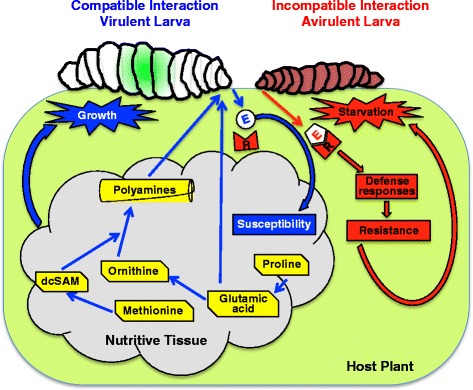
**Model depicting the involvement of polyamines in susceptibility to virulent Hessian fly larvae.** Hessian fly larvae apply effectors (E) to host cells. In the absence of effector recognition by wheat *R* gene products (R) a compatible interaction (virulent larva on susceptible plant; represented in blue) takes place resulting in the formation of a nutritive tissue that is rich in amino acids and other nutrients. Some amino acids are converted to polyamines directly, or indirectly through the mediation of decarboxylated s-adenosylmethionine (dcSAM). The polyamines and amino acids increase the nutritional value of the host tissue and are ingested by virulent Hessian fly larvae, benefiting their growth and development. In contrast, recognition of larval effectors by wheat *R* gene products triggers an incompatible interaction (avirulent larva on resistant plant; represented in red) takes place, leading to a cascade of defense responses that do not allow the formation of a nutritive tissue, and eventually result death of larvae by starvation.

The response of polyamines during biotic stress varies for different host-pathogen systems [8,64]. Contrary to other interactions where polyamines play a role in resistance, salivary elicitors from the avirulent Hessian fly larvae are promptly detected by the resistant wheat host surveillance mechanism but do not trigger polyamine production during the defense response (Figure 8). In susceptible wheat responding to virulent Hessian fly larval elicitors, a dramatic increase occurs in free polyamine levels along with amino acids and sugars, adding to the nutritional component of the plant-derived larval diet. Although the capacity of virulent larvae to convert ornithine to putrescine is limited due to low expression of the *Mdes-odc* gene, other genes in the polyamine pathway become activated, suggesting that the source of increased larval polyamine abundance is plant-derived. Further studies involving genetic manipulation of both free as well as conjugated polyamine metabolism in wheat will reveal valuable information on a more definitive biological role of these molecules during wheat-Hessian fly interactions.

## Methods

### Insect material

Hessian fly (*Mayetiola destructor*) laboratory stocks of biotype L (avirulent on wheat lines carrying the *H9* or *H13* resistance genes and virulent on susceptible ‘Newton’ wheat carrying no genes for resistance), *vH9* (virulent on ‘*H9-*Iris’ wheat), *vH13* (virulent on ‘*H13’* wheat) and biotype GP (virulent on Newton wheat) were used in the present study and maintained in diapause at 4°C cold room at the USDA-ARS Crop Production and Pest Control Research Unit, at Purdue University, as described by Foster et al. [65].

### Plant material

For transcriptional profiling studies, wheat (*Triticum aestivum*) seedlings were reared in a growth chamber using a randomized block design with replicates blocked by time or location. Three different experimental designs were used in the current study. In the first design, two nearly isogenic wheat lines *H9*-Iris (resistant, carrying the *H9* resistance gene) and Newton (susceptible, carrying no genes for resistance) were infested with biotype L flies. In the second design, *H9*-Iris wheat was infested with either *vH9* (virulent on *H9*-Iris) or biotype L (avirulent on *H9*-Iris) flies. In the third design wheat line ‘PI9346A1-2-5-5-2’ (carrying the *H13* resistance gene, and designated ‘*H13*-wheat’ in this paper) was infested with either *vH13* (virulent on *H13*-wheat) or biotype L (avirulent on *H13*-wheat) flies. Wheat-Hessian fly interactions that result in induced plant susceptibility are defined as compatible, whereas those leading to induced plant resistance are defined as incompatible.

### Plant growth and infestation

Twelve seeds were sown in each 10-cm diameter pot containing Promix Professional growing medium (Premier Horticulture Inc., Quakertown, PA, USA) and placed in a Conviron growth chamber (Controlled Environments Limited, Winnipeg, Manitoba, Canada). The growth chamber was set at 18°C with a 24-h photoperiod (irradiance between 980 and 1470 μmol m^−2^ sec^−1^), and 60% relative humidity. When the plants were at the 1-leaf stage, pots were covered with vented plastic cups and five mated female Hessian flies were introduced resulting in infestation levels of approximately 18 larvae per plant. Control uninfested plants were treated identically except no flies were released inside the cups. To confirm that infestation resulted in the correct interactions, 10 plants per treatment per replicate were dissected 8 days after Hessian fly eggs hatched (DAH) to count the number of living and dead larvae at the crown of the plant.

### Tissue collections and sample preparation

Wheat seedling samples for transcriptional profiling studies were collected from the first one-cm above the root-shoot junction of leaf 2 (leaf on which the larvae were feeding). Tissues from infested and uninfested control plants were collected over a time-course of 1, 2, 3, 4 and 8 DAH and frozen in liquid nitrogen. Samples included pooled tissues from 25–30 plants for each time-point in three biologically replicated experiments. To collect insect tissue for expression profiling studies, leaf 2 from *H9*-Iris and Newton wheat plants containing the biotype-L larvae were dissected under a microscope into deionized water to dislodge the larvae. The larvae were then pipetted into a 1.5 ml microfuge tube, flash frozen in liquid nitrogen and stored at −80°C until further use. Larval tissues were collected over a time-course of 1, 2, 3, 4 and 8 DAH from 30 plants for each time-point in three biologically replicated experiments. In addition, neonate control larvae that had not fed on plant tissue were also collected using the following method. Three days post-infestation of Newton wheat plants with mated adult female Hessian flies and just before egg hatch, mature leaves containing the eggs were cut at the ligule and placed overnight into a 100 ml beaker containing 50 ml deionized water. To prevent desiccation and to increase humidity, a larger beaker was used to cover the smaller beaker containing the leaves. Upon egg hatch, the larvae that crawled into the water were collected into a 1.5 ml microfuge tube, flash frozen in liquid nitrogen and stored at −80°C until further use. RNA from wheat and larval samples was isolated with TRIzol reagent (Invitrogen, Carlsbad, CA) according to the manufacturer’s protocol. Reverse transcription to generate cDNA for use in quantification steps was conducted as described in Subramanyam et al. [57].

### Identification of the *Mayetiola destructor* polyamine biosynthesis pathway gene sequences

Orthologous sequences of genes involved in polyamine biosynthesis were obtained for *Drosophila melanogaster* from Fly Base (www.flybase.org) [66], *Tribolium castaneum* from Beetle Base (www.beetlebase.org) [67], and *Anopheles gambiae* and *Aedes aegypti* from Vector Base (www.vectorbase.org) [68]. The recently assembled Hessian fly genome (www.agripestbase.org/hessianfly) was used to identify sequences of target genes using the tblastn program. The assembly has BLAST results linking to GBROWSE [69], a genome viewer that contains gene annotations automatically generated by gene prediction software. The generated GBROWSE annotations were saved along with flanking regions (5 kb or more) upstream and downstream of the annotated gene. These sequences were analyzed for intron/exon boundaries, as well as missing or incomplete exons that varied from known gene structure, by conducting multiple sequence alignments of protein sequence deduced from the Hessian fly genome with deduced protein sequences obtained from orthologs of other insect species.

### Cloning and sequencing of polyamine pathway genes

PCR-based gene cloning was used to determine whether Hessian fly sequences generated by automated genome annotation were partial or complete. Forward and reverse primers anchored at the predicted start and stop codons were designed (Table 1). Total RNA was isolated from 4 DAH Hessian fly biotype Great Plains larvae feeding on susceptible Newton wheat to serve as a template for cDNA synthesis using the First-Strand Synthesis System for RT-PCR (Invitrogen) with oligo-dT primers. The target sequences for cloning were amplified in a 50 μl reaction mixture containing 10 X PCR Buffer, 2 mM MgSO_4_, 0.2 μM each of gene-specific primers, 0.2 mM dNTPs, 50 ng cDNA template and 1 unit of Platinum *Taq* High Fidelity polymerase (Invitrogen). The PCR cycling parameters were 94°C for 2 min, 35 cycles of 94°C for 30s, 50°C for 30s and 68°C for 3 min, with a final extension of 68°C for 10 min. The resulting amplicons were gel-purified using the MinElute Gel Extraction Kit (Qiagen, Valencia, CA), cloned using the TOPO-TA Cloning Kit for Sequencing (Invitrogen) and sequenced at the Purdue University Genomics Core Facility. The sequences were submitted to NCBI GenBank (*Mdes-odc*: KJ136117, *Mdes-samdc*: KJ136120, *Mdes-spds*: KJ136119, *Mdes-spms*: KJ136118).

**Table 1:** **Primers for quantitative real time PCR (RT-qPCR) and for cloning of cDNA of Hessian fly transcripts from polyamine biosynthesis pathway**

**Gene**	**GenBank**	**Primer Sequence**	**Primer Sequence**
	**Accession**	**(RT-qPCR)**	**(PCR for cloning)**
ubiquitin	DQ674274	5’ cccctgcgaaaattgatga 3’	-
(*Mdes-ubq*)		5’ aaccggactacttgcatcgaa 3’	
**Polyamine biosynthesis**			
ornithine decarboxylase	KJ136117	5’ gaaccaggacgattttatgtagca 3’	5’ atgaaaatctacggatcaaataaattgc 3’
*Mdes-odc*		5’ cgaatttcacgtttcgaatgaa 3’	5’ ttagttgtcgatcaagctatcggg 3’
s-adenosylmethionine	KJ136120	5’ accccatcgcggcttt 3’	5’ atgaacaaaaacgccgacttgcggaa 3’
decarboxylase		5’ ggcccgaccattgtcaaa 3’	5’ cgtagccaggaaaacgacaatattgaa 3’
*Mdes-samdc*			
spermidine synthase	KJ136119	5’ agtgaagctcatggcaaaacg 3’	5’ atggacacaattcgaaatggttggttc 3’
*Mdes-spds*		5’ tcatccttttccgtgcattg 3’	5’ cgatgaattaagttgtttagcaattgatc 3’
spermine synthase	KJ136118	5’ ttttgggaggcggtgatg 3’	5’ atgtccgctcaaacaattctattg 3’
*Mde-spms*		5’ ggtcacgaactttggattttcttt 3’	5’ tcaacaagaattatcaaatgttatttgat 3’

Near full-length cDNA sequences for wheat genes s-adenosylmethionine decarboxylase (*samdc*) and spermidine synthase (*spds*) were cloned using BD SMART RACE kit (Clontech, Mountainview, CA) according to the manufacturer’s protocol. A 439 bp sequence encoding SAMDC and a 947 bp sequence encoding a SPDS, obtained from a suppressive subtractive cDNA library (Sardesai and Williams, unpublished data), were used as seed sequence to design primers for cloning regions extending in the 5’ direction. The 5’RACE primers for *samdc* and *spds* are given in Additional file 8: Table S2. Total RNA (1 μg) isolated from biotype L-infested *H9*-Iris wheat 1 DAH was used as the template. The resulting PCR products were then cloned into the pCR4 TOPO vector (Invitrogen) and sequenced at the Purdue University Genomics Core Facility. The cDNA sequences were submitted to NCBI GenBank (*Hfr-samdc*: HQ121401 and *Hfr-spds*: HQ121400).

### Quantitative (Real-Time) Reverse Transcription PCR analyses

Transcript profiling by quantitative (Real-time) reverse transcription PCR (RT-qPCR) utilized target-specific primers designed with Primer Express 3.0 Software from Applied Biosystems, (Foster City, CA). Primers for most wheat target genes were designed from sequences obtained from GenBank (Table 2). Primers for wheat s-adenosylmethionine decarboxylase (*Hfr-samdc*), and spermidine synthase (*Hfr-spds*) were designed from near full-length cDNA sequences cloned from *T. aestivum* cultivar Iris (Table 2). Primers for Hessian fly target genes were designed from genes annotated from the Hessian fly genome assembly (http://agripestbase.org/hessianfly) (Table 1).

**Table 2:** **Wheat gene-specific primers for quantitative real-time RT PCR (RT-qPCR)**

**Gene**	**Abbreviation**	**GenBank**	**Primer Sequence**
		**Accession**	
ubiquitin	*Ta-ubq*	X56803	5’ggtgtctccggtatcctccaa 3’
			5’ tgctccacaccagcagaagt 3’
**Polyamine biosynthesis**			
ornithine decarboxylase	*Ta-odc*	XP_003578555	5’ gctccaacttcaacggcttct 3’
			5’ cgaatggcgtgtgctacgta 3’
arginine decarboxylase	*Ta-adc*	EU236151	5’ gttgtatcgtgttactcatggtcgta 3’
			5’ gacgcatgggaaataaaaagatg 3’
s-adenosylmethionine	*Hfr-samdc*	HQ121401	5’ aggcaagctcgccaacct 3’
decarboxylase			5’ ggaatagcgacagcaaatcatg 3’
s-adenosylmethionine	*Ta-sams*	EMS47328	5’ cgtcatcggcggacctca 3’
synthetase			5’ ttggtcgggtccttgccagagaa 3’
spermidine synthase	*Hfr-spds*	HQ121400	5’ gcggtgttctttctaatctagctgaa 3’
			5’ gtgcacgccacccttgaata 3’
arginase	*Ta-arg*	CA598716	5’ gaagctgagcgcccaaga 3’
			5’ tttgttgcttcggtcctgact 3’
**Ornithine biosynthesis**			
pyrroline-5-carboxylate	*Ta-p5cs*	AFO22914	5’ gcaccctcgaatttgttgatg 3’
synthetase			5’ acaatctgtgtgtgcacttccat 3’
ornithine aminotransferase	*Ta-oat*	AFO22915	5’ ggcacggaggcaaatgag 3
			5’ agtgaaataatgtcatgggaacca 3’
pyrroline-5-carboxylate	*Ta-p5cr*	AY880317	5’ ttcccctgcaggaactacca 3’
reductase			5’ gcatcttgttgtggcagcaa 3’
glutamate reductase	*Ta-glr*	BJ219898	5’ gcgtggtggatgaaagttcttat 3’
			5’ tgccggcaacttatgatgaa 3’
acetylornithinase	*Ta-aor*	BJ321340	5’ tctgcgaggaaggcttgaa 3’
			5’ agattacaggccaccccattc 3’

The RT-qPCR was performed on an Applied Biosystems (ABI) 7500 Fast Sequence Detection System using SYBR Green master mix. Reaction volumes of 10 μl contained 5 μl of 2X SYBR Green I PCR Master Mix (ABI), gene-specific primers at a final concentration of 0.2 μM each and 20 ng of cDNA template. PCR parameters were as follows: 95°C for 10 min, 40 cycles of 95°C for 3 sec and 60°C for 30 sec. Following amplification, RT-qCR primer specificity to a single target sequence was verified through melt curve analysis. All PCRs were carried out in triplicate for each of the three biological replicates. No-template negative controls were included in each PCR plate. In addition, expression levels of constitutive wheat and Hessian fly ubiquitin genes were used as internal controls to normalize amounts of target cDNA in all samples. Relative expression values (REV) were calculated using the standard curve method (User Bulletin 2: ABI PRISM 7700 Sequence Detection System) using serial dilutions of a cDNA sample containing the target sequence [70].

### Statistical analysis of RT-qPCR data

Significant differences in the logarithm-transformed REVs were determined by analysis of variance (ANOVA) using the PROC MIXED procedure of SAS software version 9.1.3 (SAS Institute Inc.). The ANOVA model included treatments, time-points, biological replicates and the interaction between treatments and time-points as fixed effects. Data from the three biological and technical replicates were combined and included as a random effect in the analysis model. Differences were considered statistically significant if the *p-*value associated with the contrast was less than 0.05. All *p*-values were adjusted using Bonferroni correction. The data are presented as log fold-change so that large and small changes could be represented on one graph. Transcript levels in infested wheat plants were compared to levels in uninfested controls at the same time-point. Transcript levels in virulent and avirulent Hessian fly larvae (feeding on wheat plants) were compared to levels in neonate larvae that had never fed on the host wheat.

### Polyamine analysis

The wheat crown tissue (one-cm segments from leaf sheath 2) was collected from both infested (biotype L infested *H9*-Iris and Newton) and uninfested control plants over a time course (4, 6, and 8 DAH) as described previously. Larval samples (biotype L feeding on *H9*-Iris and Newton) on leaf sheath 2 were collected over a time course (4, 6, and 8 DAH) as described previously. 100–200 mg of plant or 50–100 mg of larval tissues were mixed with four volumes of 5% perchloric acid. The samples were then frozen (20°C) and thawed (room temperature) three times before dansylation and quantification of polyamines by HPLC [71]. The dansyl-polyamines were dissolved in 1 ml methanol and separated on a reversed-phase C18 column for HPLC (Perkin Elmer, Waltham, MA) using a gradient of acetonitrile (40-100%) and 10 mM heptanesulfonic acid, pH 3.4. A fluorescence detector (Perkin Elmer) was used for quantification and the polyamine concentrations were expressed as nmol (gFW)^−1^. Significant differences in polyamine levels were determined by SAS as described previously. Polyamine levels in infested wheat plants were compared to levels in uninfested controls, whereas polyamines levels in virulent larvae were compared to levels in the avirulent larvae at the same time-points.

### Analyses of SAMDC activity in wheat crown tissue

Crown tissue (leaf sheath 2) from uninfested and biotype L-infested wheat lines, *H9*-Iris and Newton (20–30 plants), was harvested as described previously over a time-course, 4, 6 and 8 DAH. The activity of SAMDC (EC 4.1.1.50) was measured in the tissue extracts using radiolabeled substrates as described by Minocha et al. [72]. 50 μL of the labeled substrate, a solution of 0.1 μCi of [1-^14^C-SAM], specific activity 58 mCi mmol^−1^ (ARC, Inc. St. Louis, MO) plus 4 mM unlabeled SAM were added to 200–400 mg of each sample. The decarboxylation of SAM by the plant SAMDC generated ^14^CO_2_ that was adsorbed onto Whatman 3 MM filter paper soaked with 50 μL Scintigest (Fisher Scientific, Pittsburgh, PA) and quantified in 10 mL of Scintilene (Fisher Scientific) in an LSC-6000 liquid scintillation counter (Beckman, Fullerton, CA). SAMDC activity is expressed as nmol CO_2_ (gFW) ^-1^(h)^−1^. Significant difference in SAMDC activity was determined by SAS as described previously. Differences in the activity were compared between infested wheat plants and the uninfested control at the same time-point.

### Treatment of wheat plants with D,L-α-Difluoromethylornithine (DFMO)

The influence of plant polyamines on growth of virulent Hessian fly larvae was investigated by applying DFMO (Sigma, St. Louis, MO) at three concentrations (1, 3 or 5 mM with 0.01% Tween 20) to susceptible Newton wheat to inhibit the activity of the enzyme, Ta-ODC that catalyzes the conversion of ornithine to putrescine. At the 2-leaf stage, the first leaf was individually brushed with a thin coating of desired concentration of DFMO and allowed to dry before infestation with virulent biotype L Hessian fly 24 h later. 15 plants were treated with each DFMO concentration. Control plants (15) were brushed with only 0.01% Tween 20 prior to infestation. The eggs were laid on leaf blade 2 and larvae set up feeding sites on leaf sheath 3; thus the insects were never in direct contact with the DFMO that was applied on leaf 1of the plants. Photomicrographs of the larvae from 10 plants were taken 7 DAH and larval lengths measured. To assess developmental delays, number of larvae and pupae on DFMO-treated and untreated plants were counted (5 plants per sample) 18 DAH. Statistical analyses of larval lengths and rate of development were carried out by one-way ANOVA using SAS. Differences were considered statistically significant if the *p-*value was <0.05.

### Phylogenetic analyses

The sequences of *M. destructor* genes, which were cloned and used for expression studies, were verified as belonging to the polyamine biosynthesis pathway by comparing their evolutionary relationships, through phylogenetic tree construction, with orthologs identified from other insect genomes (*T. castaneum*, *B. mori*, *D. melanogaster*, *A. gambiae*, *A. aegypti*). The peptide sequences for the genes annotated were aligned with their respective groups using the ClustalX program, version 2.1 [73]. A phylogenetic tree was constructed with the MrBayes 3 [74] program using maximum likelihood analyses run for 1×10^6^ generations with a burn-in of 25%. Split frequency deviations were less than or equal to 0.01, and posterior probabilities from the majority consensus rule were reported. The phylogenetic tree was displayed using TreeView [75].

### Availability of supporting data

All the data supporting our results are included in the article and in the Additional files.

## Additional files

Additional file 1:
**Abundance of polyamine biosynthesis pathway transcripts in** ***H9-Iris*** **wheat infested with biotype L (avirulent) and** ***vH9*** **(virulent) Hessian fly larvae.** Transcript levels of a) *Ta-odc*, b) *Hfr-spds,* c) *Ta-sams,* and d) *Hfr-samdc* from crown tissue (leaf 2) quantified by RT-qPCR. Values are the log fold-change ± SE of infested compared to the uninfested plants (baseline of 0). Statistically significant (*p* < 0.05) differences are indicated by ‘*’ with linear fold-change values. Additional file 2:
**Abundance of polyamine biosynthesis pathway transcripts in** ***H13-*** **wheat infested with biotype L (avirulent) and** ***vH13*** **(virulent) Hessian fly larvae.** Transcript levels of a) *Ta-odc*, b) *Hfr-spds*, c) *Ta-sams,* and d) *Hfr-samdc* from crown tissue (leaf 2) quantified by RT-qPCR. Values are the log fold-change ± SE of infested compared to the uninfested plants. Statistically significant (*p* < 0.05) differences are indicated by ‘*’ with linear fold-change values. Additional file 3:
**Abundance of ornithine biosynthesis pathway transcripts in** ***H9-Iris*** **and Newton wheat infested with biotype L Hessian fly larvae.** Transcript levels of a) *Ta-p5cr*, b) *Ta-arg*, and c) *Ta-oat* from crown tissue (leaf 2) quantified by RT-qPCR*.* Values are the log fold-change ± SE of infested compared to the uninfested plants. Statistically significant (*p* < 0.05) differences are indicated by ‘*’ with linear fold-change values. Additional file 4:
**Abundance of ornithine biosynthesis pathway transcripts in** ***H9-Iris*** **wheat infested with biotype L (avirulent) and** ***vH9*** **(virulent) Hessian fly larvae.** Transcript levels of a) *Ta-p5cs*, b) *Ta-oar*, c) *Ta-arg,* d) *Ta-glr,* e) *Ta-p5cr,* and f) *Ta-oat*. Values are the log fold-change ± SE of infested plants compared to the uninfested plants. Statistically significant (*p* < 0.05) differences are indicated by ‘*’ with linear fold-change values. Additional file 5:
**Abundance of ornithine biosynthesis pathway transcripts in** ***H13-*** **wheat infested with biotype L (avirulent) and** ***vH13*** **(virulent) Hessian fly larvae.** Transcript levels of a) *Ta-p5cs*, b) *Ta-oar,* c) *Ta-arg,* d) *Ta-glr*, e) *Ta-p5cr,* and f) *Ta-oat*. Values are the log fold-change ± SE of infested plants compared to the uninfested plants. Statistically significant (*p* < 0.05) differences are indicated by ‘*’ with linear fold-change values. Additional file 6: Table S1. Contains annotation of polyamine biosynthesis pathway genes from Hessian fly genome. Additional file 7:
**Bayesian maximum likelihood dendrogram.** A phylogenetic tree generated from deduced amino acid sequence alignments of genes annotated from the Hessian fly, *Mayetiola destructor* genome, for the polyamine biosynthesis pathway with orthologous sequences from *Tribolium castaneum*, *Bombyx mori*, *Drosophila melanogaster*, *Aedes aegypti*, and *Anopheles gambiae*. Posterior probability values are located at the nodes. Sequences for the genes annotated from Hessian fly group with their respective analogous sequences from other insect species. Characters in parentheses indicate GenBank accession numbers. The scale bar represents 0.1 substitutions per nucleotide site. Additional file 8: Table S2. Contains the sequences of primers for carrying out 5’RACE.
