# Supplementary figures and images for: Hessian fly larval feeding triggers enhanced polyamine levels in susceptible but not resistant wheat
Source: BMC Plant Biol. 2015 Jan 16;15:3. doi: 10.1186/s12870-014-0396-y (PMC4308891; doi:10.1186/s12870-014-0396-y)

Additional file 1

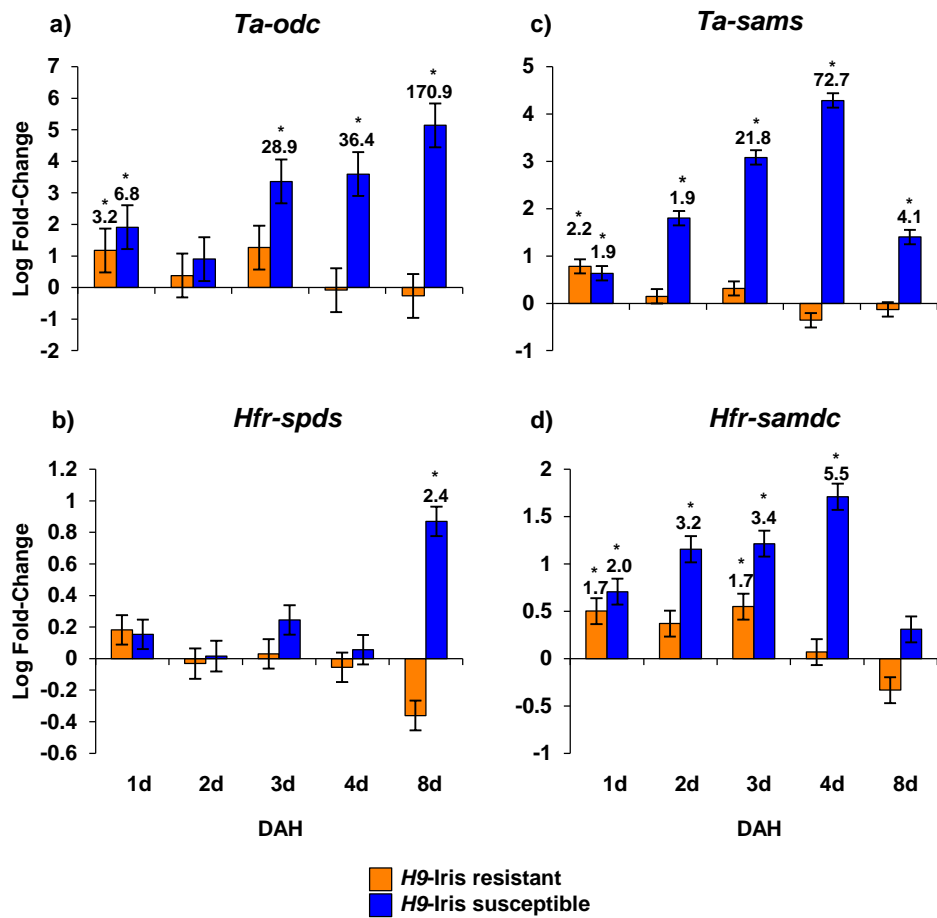

Supplement: Additional file 1: — Abundance of polyamine biosynthesis pathway transcripts in H9-Iris wheat infested with biotype L (avirulent) and vH9 (virulent) Hessian fly larvae. Transcript levels of a) Ta-odc, b) Hfr-spds, c) Ta-sams, and d) Hfr-samdc from crown tissue (leaf 2) quantified by RT-qPCR. Values are the log fold-change ± SE of infested compared to the uninfested plants (baseline of 0). Statistically significant (p < 0.05) differences are indicated by ‘*’ with linear fold-change values. [file 12870_2014_396_MOESM1_ESM.pdf]

Additional file 2

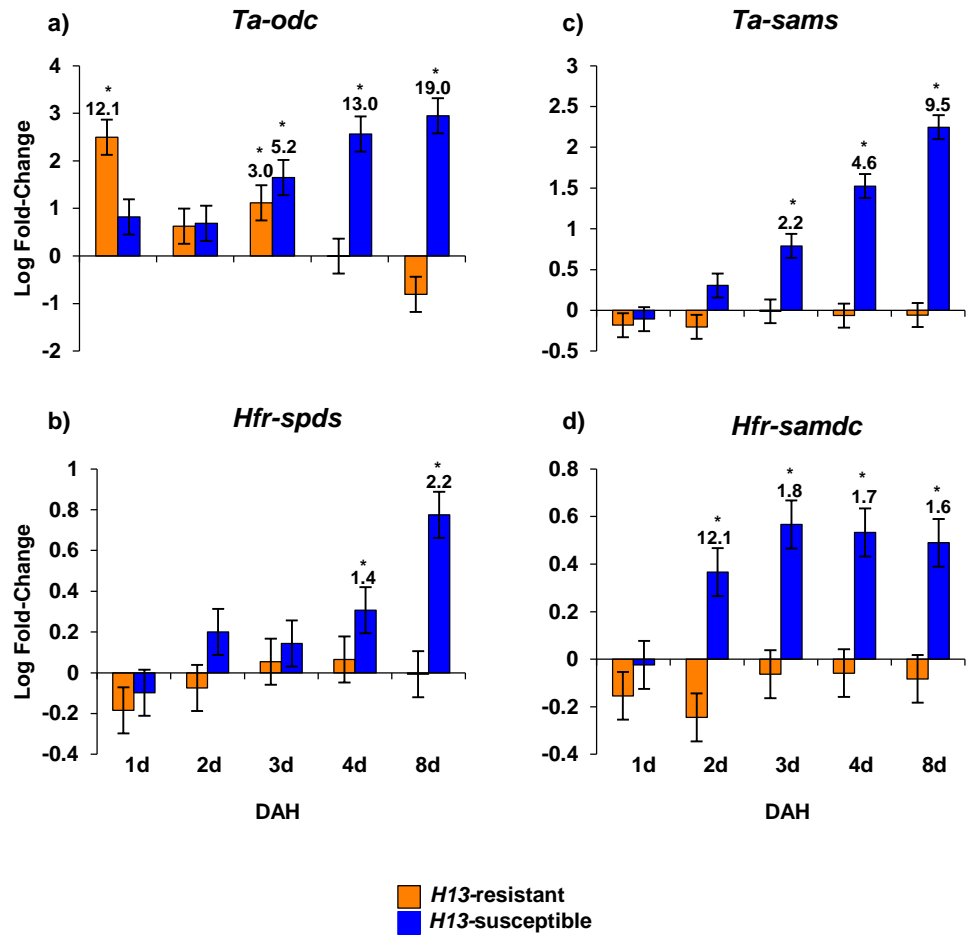

Supplement: Additional file 2: — Abundance of polyamine biosynthesis pathway transcripts in H13- wheat infested with biotype L (avirulent) and vH13 (virulent) Hessian fly larvae. Transcript levels of a) Ta-odc, b) Hfr-spds, c) Ta-sams, and d) Hfr-samdc from crown tissue (leaf 2) quantified by RT-qPCR. Values are the log fold-change ± SE of infested compared to the uninfested plants. Statistically significant (p < 0.05) differences are indicated by ‘*’ with linear fold-change values. [file 12870_2014_396_MOESM2_ESM.pdf]

Additional file 3

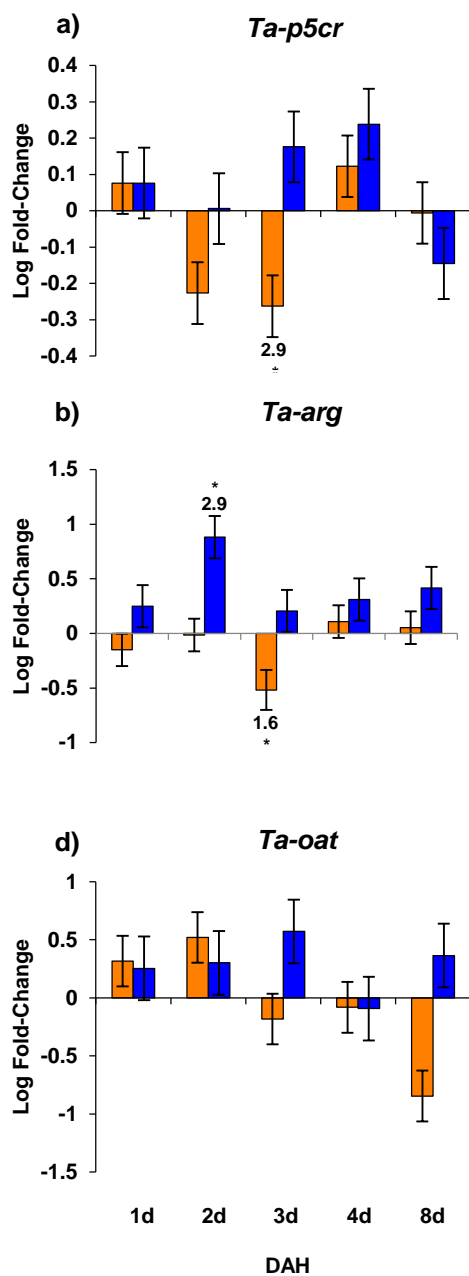

Supplement: Additional file 3: — Abundance of ornithine biosynthesis pathway transcripts in H9-Iris and Newton wheat infested with biotype L Hessian fly larvae. Transcript levels of a) Ta-p5cr, b) Ta-arg, and c) Ta-oat from crown tissue (leaf 2) quantified by RT-qPCR. Values are the log fold-change ± SE of infested compared to the uninfested plants. Statistically significant (p < 0.05) differences are indicated by ‘*’ with linear fold-change values. [file 12870_2014_396_MOESM3_ESM.pdf]

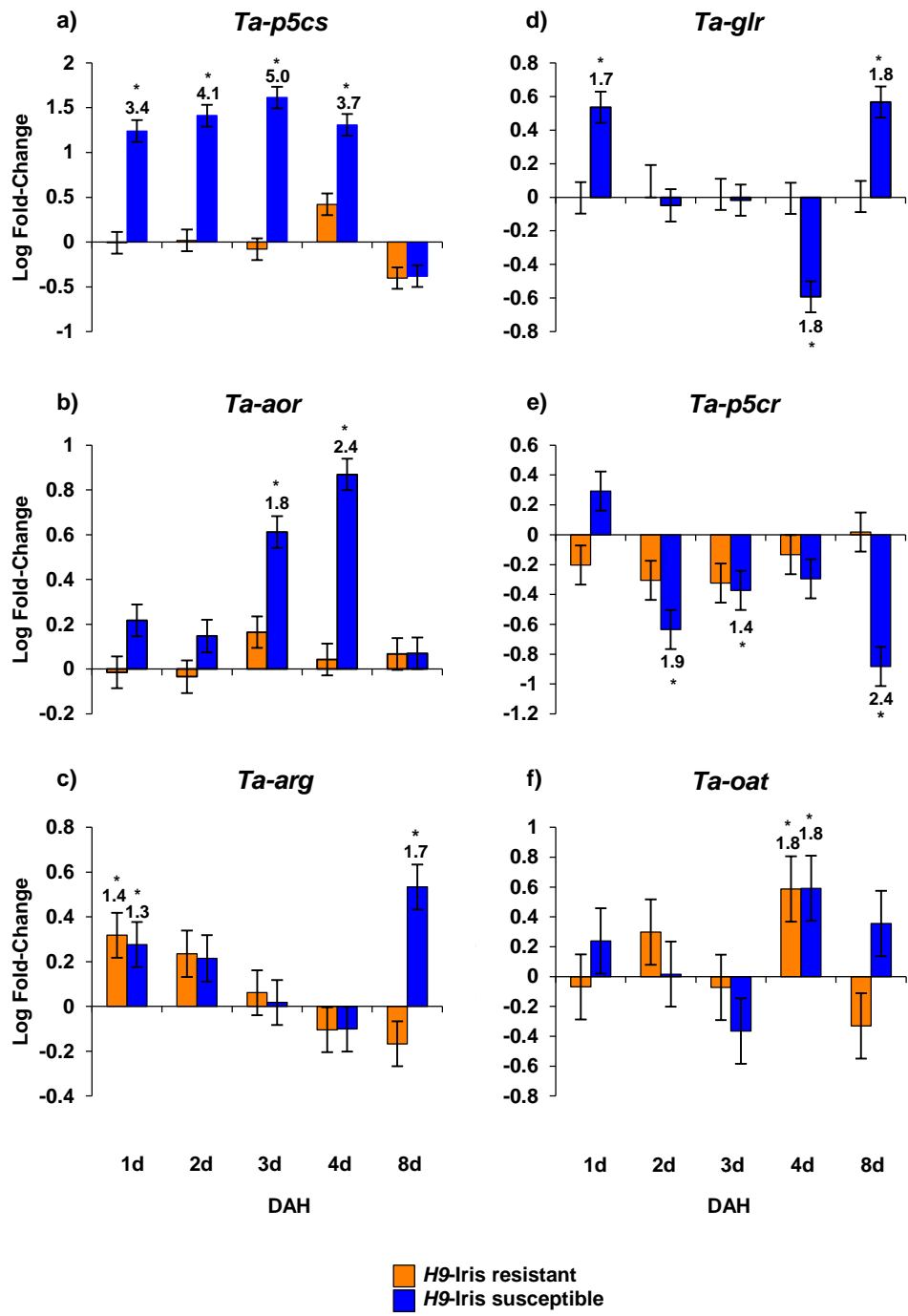

Supplement: Additional file 4: — Abundance of ornithine biosynthesis pathway transcripts in H9-Iris wheat infested with biotype L (avirulent) and vH9 (virulent) Hessian fly larvae. Transcript levels of a) Ta-p5cs, b) Ta-oar, c) Ta-arg, d) Ta-glr, e) Ta-p5cr, and f) Ta-oat. Values are the log fold-change ± SE of infested plants compared to the uninfested plants. Statistically significant (p < 0.05) differences are indicated by ‘*’ with linear fold-change values. [file 12870_2014_396_MOESM4_ESM.pdf]

Additional file 5

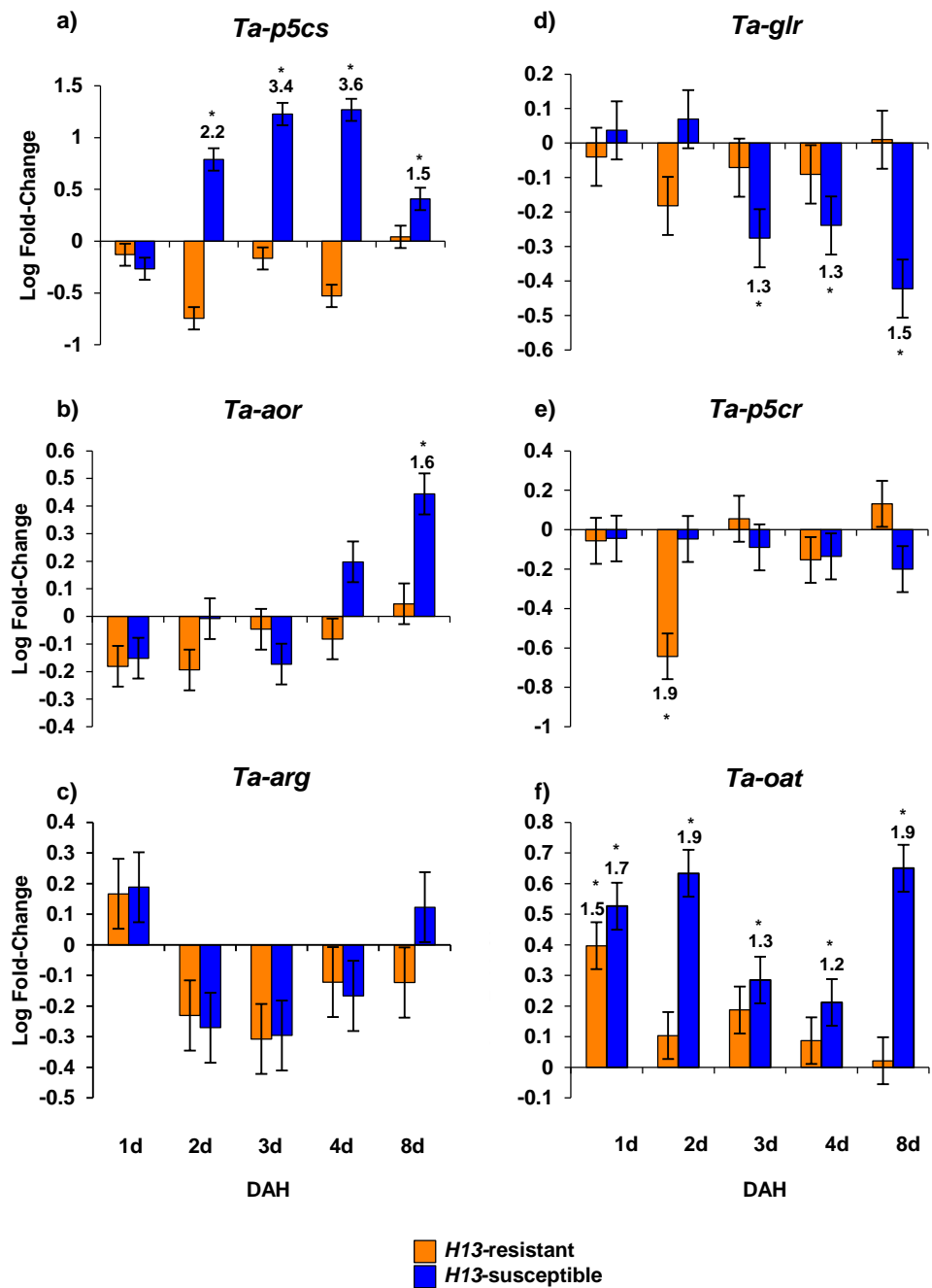

Supplement: Additional file 5: — Abundance of ornithine biosynthesis pathway transcripts in H13- wheat infested with biotype L (avirulent) and vH13 (virulent) Hessian fly larvae. Transcript levels of a) Ta-p5cs, b) Ta-oar, c) Ta-arg, d) Ta-glr, e) Ta-p5cr, and f) Ta-oat. Values are the log fold-change ± SE of infested plants compared to the uninfested plants. Statistically significant (p < 0.05) differences are indicated by ‘*’ with linear fold-change values. [file 12870_2014_396_MOESM5_ESM.pdf]

## Additional file 7

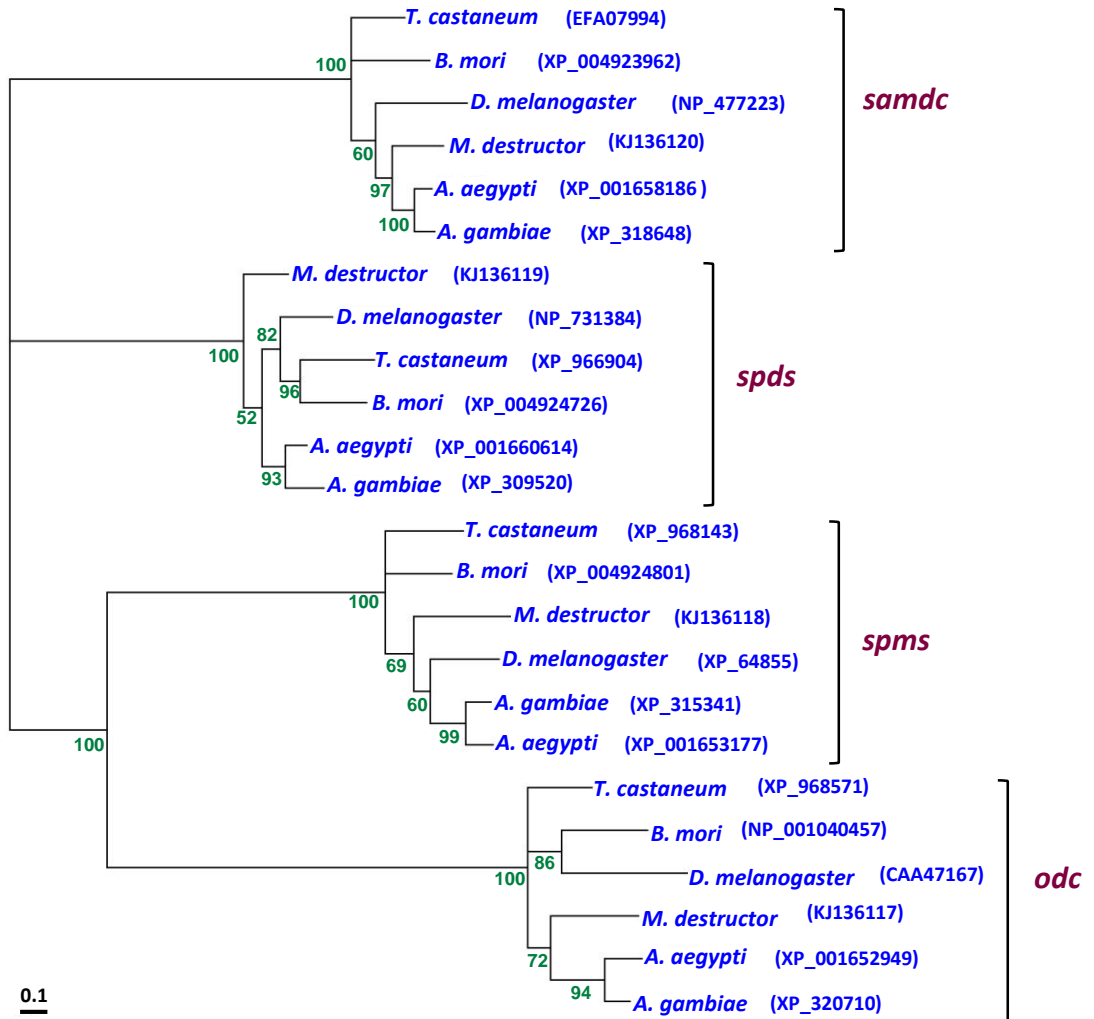

Supplement: Additional file 7: — Bayesian maximum likelihood dendrogram. A phylogenetic tree generated from deduced amino acid sequence alignments of genes annotated from the Hessian fly, Mayetiola destructor genome, for the polyamine biosynthesis pathway with orthologous sequences from Tribolium castaneum, Bombyx mori, Drosophila melanogaster, Aedes aegypti, and Anopheles gambiae. Posterior probability values are located at the nodes. Sequences for the genes annotated from Hessian fly group with their respective analogous sequences from other insect species. Characters in parentheses indicate GenBank accession numbers. The scale bar represents 0.1 substitutions per nucleotide site. [file 12870_2014_396_MOESM7_ESM.pdf]
