# Supplementary material for: Hessian fly larval feeding triggers enhanced polyamine levels in susceptible but not resistant wheat
Source: BMC Plant Biol. 2015 Jan 16;15:3. doi: 10.1186/s12870-014-0396-y (PMC4308891; doi:10.1186/s12870-014-0396-y)
Supplement: Additional file 6: Table S1. — Contains annotation of polyamine biosynthesis pathway genes from Hessian fly genome. [file 12870_2014_396_MOESM6_ESM.docx]

**Table S1.** Annotation of polyamine biosynthesis pathway genes from Hessian fly genome

**Gene Chromosome E-value Blast Hit*** (**Scaffold position) (*A. aegypti*)**

**______________________________________________________________________________________**

*Mdes-odc* X1.1 8e-161

*Mdes-samdc* A1.47 8e-145

*Mdes-spds* A2.1 2e-153

*Mdes-spms* X1Random.8 9e-137

*Hessian fly genes showed significant blast hit with orthologs from *A. aegypti*
