# Supplementary material for: Hessian fly larval feeding triggers enhanced polyamine levels in susceptible but not resistant wheat
Source: BMC Plant Biol. 2015 Jan 16;15:3. doi: 10.1186/s12870-014-0396-y (PMC4308891; doi:10.1186/s12870-014-0396-y)
Supplement: Additional file 8: Table S2. — Contains the sequences of primers for carrying out 5’RACE. [file 12870_2014_396_MOESM8_ESM.docx]

**Table S2.** Primers for 5’RACE

**______________________________________________________________________________**

**Gene Abbreviation Primer Sequence**

**______________________________________________________________________________**

s-adenosylmethionine *Hfr-samdc*  5’ gtcttccactgcttcgaggccgaga 3’

decarboxylase

spermidine synthase *Hfr-spds*  5’ ttcaagggtggcgtgcactatgcctgggca 3’

**______________________________________________________________________________**
